# Circulating Levels of the Short-Chain Fatty Acid Acetate Mediate the Effect of the Gut Microbiome on Visceral Fat

**DOI:** 10.3389/fmicb.2021.711359

**Published:** 2021-07-15

**Authors:** Ana Nogal, Panayiotis Louca, Xinyuan Zhang, Philippa M. Wells, Claire J. Steves, Tim D. Spector, Mario Falchi, Ana M. Valdes, Cristina Menni

**Affiliations:** ^1^Department of Twin Research and Genetic Epidemiology, King’s College London, London, United Kingdom; ^2^Nottingham NIHR Biomedical Research Centre at the School of Medicine, Nottingham City Hospital, University of Nottingham, Nottingham, United Kingdom

**Keywords:** acetate, *Lachnoclostridium*, *Coprococcus*, human gut microbiota, visceral fat

## Abstract

**Background:**

Acetate is a short-chain fatty acid (SCFA) produced by gut bacteria, which has been implicated in cardio-metabolic health. Here we examine the relationships of circulating acetate levels with gut microbiome composition and diversity and with visceral fat in a large population-based cohort.

**Results:**

Microbiome alpha-diversity was positively correlated with circulating acetate levels (Shannon, Beta [95%CI] = 0.12 [0.06, 0.18], *P* = 0.002) after adjustment for covariates. Six serum acetate-associated bacterial genera were also identified, including positive correlations with *Coprococcus*, *Barnesiella*, *Ruminococcus*, and *Ruminococcaceae NK4A21* and negative correlations were observed with *Lachnoclostridium* and *Bacteroides.* We also identified a correlation between visceral fat and serum acetate levels (Beta [95%CI] = −0.07 [−0.11, −0.04], *P* = 2.8 × 10^–4^) and between visceral fat and *Lachnoclostridium* (Beta [95%CI] = 0.076 [0.042, 0.11], *P* = 1.44 × 10^–5^). Formal mediation analysis revealed that acetate mediates ∼10% of the total effect of *Lachnoclostridium* on visceral fat. The taxonomic diversity showed that *Lachnoclostridium* and *Coprococcus* comprise at least 18 and 9 species, respectively, including novel bacterial species. By predicting the functional capabilities, we found that *Coprococcus* spp. present pathways involved in acetate production and metabolism of vitamins B, whereas we identified pathways related to the biosynthesis of trimethylamine (TMA) and CDP-diacylglycerol in *Lachnoclostridium* spp.

**Conclusions:**

Our data indicates that gut microbiota composition and diversity may influence circulating acetate levels and that acetate might exert benefits on certain cardio-metabolic disease risk by decreasing visceral fat. *Coprococcus* may play an important role in host health by its production of vitamins B and SCFAs, whereas *Lachnoclostridium* might have an opposing effect by influencing negatively the circulating levels of acetate and being involved in the biosynthesis of detrimental lipid compounds.

## Introduction

Acetate is a short-chain fatty acid (SCFA) produced by colonic bacteria through the saccharolytic fermentation of fibres (e.g., resistant starch, polysaccharides and simple sugars), which escape digestion and absorption (Topping and Clifton, 2001). The molar ratio of acetate in the colon is three times larger than that of the two other major SCFAs, butyrate and propionate (Cummings et al., 1987). Enteric bacteria, including *Ruminococcus* spp., *Prevotella* spp., *Bifidobacterium* spp., and *Akkermansia muciniphila* are suggested to be the main acetate-producing bacteria (Rey et al., 2010).

Recently SCFAs have received increasing attention as they have been shown to play an important role in cardio-metabolic diseases (CMD), including obesity, type-2 diabetes (T2D), arterial stiffness and atherosclerosis (Den Besten et al., 2013). Once these bacteria-derived metabolites are synthetised, they have the capacity to reach different systematic tissues, improving the gut barrier integrity, glucose, cholesterol and lipid metabolism, and regulating the immune system and anti-inflammatory response, energy intake, and blood pressure (Martin-Gallausiaux et al., 2020). For instance, acetate was shown to decrease appetite by impacting directly on the hypothalamus (Frost et al., 2014), inhibit endogenous lipolysis (Hron et al., 1978), enhance hepatic uptake of blood cholesterol (Zhao Y. et al., 2017) and reduce hyperglycaemia (Sakakibara et al., 2006). However, to gain further insight into the host-microbial cross-talk involving circulating acetate levels and its implications in cardio-metabolic health (CMH), it is important to integrate different types of data.

In this study, we analyzed the associations between circulating acetate levels, gut microbiome composition and diversity and visceral fat in a cohort of 948 women from TwinsUK. Furthermore, by performing genomic analyses, we have explored the phylogenetic diversity and metabolic complexity of the acetate-associated gut genera.

## Materials and Methods

### Study Subjects

**FIGURE 1:**
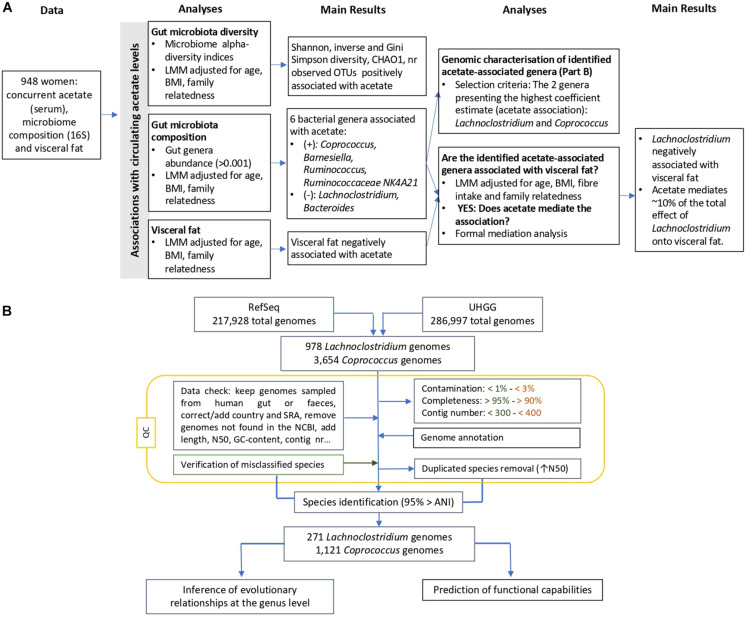
Overview of the flowchart **(A)** integrating gut microbiota composition and diversity, visceral fat and circulating acetate levels, and **(B)** showing the applied filters and conducted steps to genomically characterize the *Lachnoclostridium* spp. and *Coprococcus* spp. Steps exclusively applied for *Lachnoclostridium* and *Coprococcus* are indicated in orange and green, respectively, whereas the rest of steps were conducted in the genomes from both species. ANI, average nucleotide identity; LMM, linear mixed model; UHGG, Unified Human Gastrointestinal Genome; QC, quality control.

Study subjects were female twins enrolled in the TwinsUK registry, a national register of adult twins recruited as volunteers without selecting for any particular disease or trait (Moayyeri et al., 2013). In this study, we analyzed data from 948 female twins with concurrent measures of 16S gut microbiome composition, serum acetate levels and visceral fat. The study was approved by NRES Committee London–Westminster, and all twins provided informed written consent. A flowchart of the study design is presented in Figure 1A.

### Measurements

#### Microbiome Analysis

Fecal samples were collected and the composition of the gut microbiome was determined by 16S rRNA gene sequencing carried out as previously described (Goodrich et al., 2016). Briefly, the V4 region of the 16S rRNA gene was amplified and sequenced on Illumina MiSeq. 16S sequences were demultiplexed in QIIME 1 (Caporaso et al., 2010). The following analyses were conducted in RStudio version 1.3.1093. Amplicon sequence variants (ASV) were then generated using the “DADA2” R package following the pipeline described by Wells and colleagues (Wells et al., 2020). The ASVs were grouped into genera and the samples with less than 10,000 reads were discarded. The indices of microbiome alpha-diversity, quantified as Shannon, inverse Simpson, Gini Simpson diversity, CHAO1 and number of observed ASVs were calculated using the “microbiome” package (Lahti and Shetty, 2018).

#### Acetate Measure

Circulating levels of acetate were measured from serum by Nightingale Health Ltd. (Helsinki, Finland; previously known as Brainshake Ltd.) using a targeted NMR spectroscopy platform that has been extensively applied for biomarker profiling in epidemiological studies (Würtz et al., 2015) as previously described (Barrios et al., 2018).

#### Visceral Fat Measure

Measurements of whole body composition were performed for 948 female twins aged 48 to 87 years using the DXA fan-beam technology (Hologic QDR; Hologic, Inc., Waltham, MA, United States) as was indicated by Menni and colleagues (Menni et al., 2016). This DXA-based measurement has been validated against VF measured by CT scan (Kaul et al., 2012) and shown to be reliable and reproducible.

Briefly, subjects were positioned in a supine position wearing only a gown. The DXA machine was calibrated following the manufacturer’s suggestions. The scans were analyzed using the QDR System Software v12.6. Regions of interest were defined manually by the same operator following the SOP (derived from the manufacturer’s guidelines). The lower and upper horizontal margins were placed just above the iliac crest and at the half of the distance between the acromions and the iliac crest, respectively. The vertical margins were adjusted at the external body borders so that all the soft tissue was included.

#### Fibre Intake

A validated 131-item semi-quantitative Food Frequency Questionnaire (FFQ) established for the EPIC (European Prospective Investigations into Cancer and Nutrition)-Norfolk study (Bingham et al., 2001) was used to assess dietary intake. Estimated intakes of fiber (in grams per day) were derived from the UK Nutrient Database (McCance and Widdowson, 2014) and were adjusted for energy intake using the residual method prior to analysis (Willett and Stampfer, 1986).

### Statistical Analyses

Statistical analyses were conducted in RStudio version 1.3.1093. We assessed the association between circulating acetate and (i) indices of alpha-diversity (Shannon, inverse Simpson, Gini Simpson, CHAO1, and number of observed OTUs), (ii) gut bacterial genera abundance (genera with abundance >0.001), (iii) visceral fat using linear mixed model adjusting for age, BMI, family relatedness and multiple testing using false discovery rate [Benjamini and Hochberg (Thissen et al., 2002)]. Indices of alpha diversity were also adjusted for sequencing depth. Then, linear mixed models were further employed to investigate the association between visceral fat and any acetate-associated genera. All variables included in the models were Z-score normalized.

Finally, we employed mediation analysis as implemented in the R package “mediation” (Tingley et al., 2014) with 1,000 Monte Carlo draws for a quasi-Bayesian approximation, to test the mediation effects of acetate (indirect effect) on the total effect of *Lachnoclostridium* on visceral fat adjusting for BMI, age and fiber intake. We constructed a mediation model to quantify both the direct effect *Lachnoclostridium* on visceral fat and the indirect (mediated) effects mentioned above. The variance accounted for (VAF) score, which represents the ratio of indirect-to-total effect and determines the proportion of the variance explained by the mediation process, was further used to determine the significance of mediation effect.

### Genomic Characterization of the Identified Acetate-Associated Gut Genera

A flowchart of the steps conducted for the genomic characterisation is presented in Figure 1B.

#### Selection of Genome Sequences and Preliminary Filtering

Genomes belonging to the acetate-associated gut genera (*Lachnoclostridium* and *Coprococcus)* and their corresponding metadata were obtained from the UHGG catalog and RefSeq dataset (January, 2021), respectively (Almeida et al., 2020). We removed the RefSeq genomes derived from metagenomes and not sampled from human faeces, stool or the gastrointestinal tract, Inconsistencies related to the variable country were corrected and the missing sample accessions were added. Genomes from sample identifiers not found in the National Center for Biotechnology Information (NCBI) (Sayers et al., 2019) were discarded. The two datasets were merge and we then filtered by completeness, contamination and number of contigs (>90%, <3%, and <400 for *Lachnoclostridium* and >95%, <1%, and <300 for *Coprococcus*). The thresholds in *Lachnoclostridium* were less strict due to the scarcity of genomes presenting higher standards. Duplicated genomes were discarded, keeping the one with the highest N50 value. In total, we downloaded 271 *Lachnoclostridium* and 1,121 *Coprococcus* high-quality genomes (Supplementary Table 1). Finally, genomes from uncharacterized species or misclassified species were renamed based on the cluster given by fastANI classification (see section “Materials and Methods”).

#### Quality Assessment of Genome Assemblies and Genome Annotation

Completeness and contamination were estimated with CheckM version 1.1.3 (Parks et al., 2015) using the “lineage_wf” workflow. QUAST version 5.0.2 (Gurevich et al., 2013) was run to retrieve the total length, GC-content, contig number and N50. Genome annotation was performed using Prokka version 1.12 (Seemann, 2014) using the default parameters.

#### Average Nucleotide Identity-Based Taxonomic Classification

FastANI version 1.32 (Jain et al., 2018) was separately run on *Lachnoclostridium* and *Coprococcus* genomes to calculate the average nucleotide identity (ANI) between all pairs of sequences (Supplementary Tables 2, 3, 4). Results were filtered by the alignment fraction (>0.4), and symmetric pairwise ANI dissimilarities (100-95, ANI = 95%) were calculated from the ANI values to construct a dendrogram for each genus using the single linkage hierarchical clustering method [“hclust” R function, stats package (R Core Team and DC, 2019)]. Two networks analyses based on the information given by the dendrograms were conducted using the “layoutwithdrl” layout implemented in the “igraph” R package (Csardi and Nepusz, 2006) with an expansion and simmer attraction of 0, and an innit, liquid and crunch temperature of 100, 50, and 50, respectively.

#### Verification of Misclassified *Coprococcus* Species

The inconsistencies in the taxonomic classification were verified using BLASTn. For that, barrnap v0.9 was run to predict the 16S rRNA sequences of genomes from *C. eutactus, C. sp. BIOML-A2, C. sp. BIOML-A1, C. sp. NSJ-10*, *C. sp900066115*, and *C. sp000154*245. These were used as query and subject to perform a BLASTn search. The matches were filtered by 99% of identity and a query cover of 50%.

#### Phylogeny Inference at the Genus Level

Evolutionary relationships among the *Coprococcus* and *Lachnoclostridium* species were inferred using ezTree version 0.1 (Wu, 2018). For each species, up to three genomes (depending on the number of available genomes) sequenced from isolates were used as input. If genomes sequenced from isolates were not available, then the metagenome-assembled genomes (MAGs) with the highest completeness percentage were selected.

#### Prediction of the Functional Capabilities of *Coprococcus* spp. and *Lachnoclostridium* spp.

Metabolic Pathway Database (Metacyc) (Caspi et al., 2018) and Kyoto Encyclopedia of Genes and Genomes (KEGG) (Kanehisa et al., 2014) information for each genome was retrieved using the enzyme commission numbers from the gff files generated by Prokka and MinPath (Minimal set of Pathways) (Ye and Doak, 2009; Supplementary Table 5). For *Coprococcus* spp, only the KEGG and MetaCyc pathways related to metabolism, and fermentation, biosynthesis and degradation, respectively, were kept. *C. sp6* was not included in the analyses due to its scarcity of genomes (*n* = 1). The retrieved information was utilized to construct heatmaps [“Heatmap” R function implemented in the “ComplexHeatmap” package (Gu et al., 2016)] showing the genome percentage of each species with a given pathway. For KEGG data, only the highly different pathways between species were selected (for a given pathway, at least one species has a percentage <5% and another species has a percentage >80%). Moreover, a principal component analysis (PCA) was performed using the presence/absence matrix with the MetaCyc biosynthesis/degradation pathways using the “prcomp” R function within the “stats” package. For the three major species of *Lachnoclostridium* (species with >15 genomes), only the MetaCyc pathways related to the lipid metabolism were selected and utilized to construct a heatmap as previously indicated.

## Results

### Associations Between Circulating Acetate Levels, Gut Microbiota Composition and Diversity and Visceral Fat

The descriptive characteristics of the study participants are depicted in Table 1. Overall, 948 women were included, aged between 48 and 87 years, with an average BMI of 26.2 km/m^2^ (SD = 4.9) and concurrent measures of serum acetate levels, 16S microbiome data and visceral fat.

**TABLE 1:** Descriptive characteristics of the study population.

Phenotype	*N*	%
N	948	
Females	948	100

	**Mean**	**SD**

Age, years	65	7.84
BMI, km/m^2^	26.25	4.90
Acetate, mmol/l (log)	−0.745	0.594
Fiber intake, gr	20.3	5.70
Visceral fat, gr	613	294
**Indices of microbiome alpha-diversity**		
Shannon diversity	3.8	0.505
CHAO1	230	67.3
Number of observed OTUs	224	64.5
Inverse Simpson diversity	23.1	12.2
Gini Simpson diversity	0.938	0.05

**FIGURE 2:**
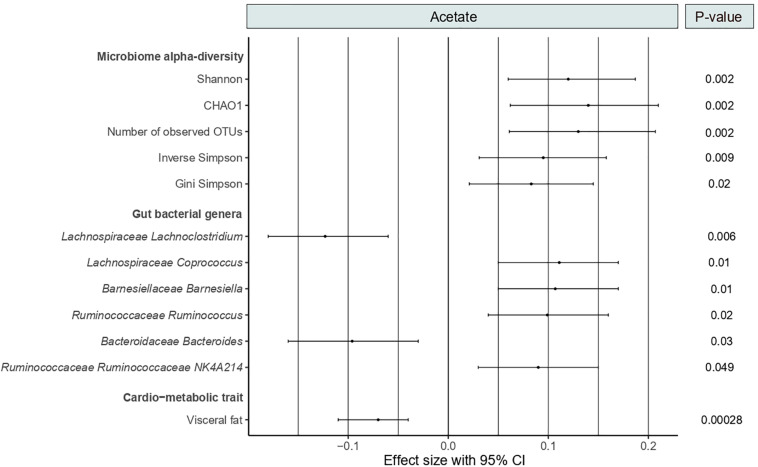
Forest plot showing the significant associations of acetate with microbiome alpha-diversity, gut bacterial genera and visceral fat. *P*-values are FDR-adjusted.

As shown in Figure 2, circulating acetate levels were positively correlated with several measures of microbiome alpha-diversity, including Shannon (Beta [95%CI] = 0.12 [0.06, 0.18], *P* = 0.002), CHAO1 (Beta [95%CI] = 0.14 [0.06, 0.21], *P* = 0.002), number of observed OTUs (Beta [95%CI] = 0.13 [0.06, 0.21], *P* = 0.002), inverse Simpson (Beta [95%CI] = 0.095 [0.03, 0.16], *P* = 0.009) and Gini Simpson (Beta [95%CI] = 0.083 [0.021, 0.15], *P* = 0.02). We then examined the association between acetate and bacterial genera abundances (genera with abundance >0.001). We identified six genera significantly associated with acetate levels after adjusting for age, BMI, family relatedness and multiple testing using FDR correction (FDR < 0.05) (Figure 2). These include *Coprococcus, Barnesiella*, *Ruminococcus*, and *Ruminococcaceae NK4A214* positively associated with acetate levels and two genera negatively associated, namely, *Lachnoclostridium* and *Bacteroides.* Among them, *Lachnoclostridium* presented the most robust association (*P* = 0.006).

As SCFAs exert benefits on CMH, we tested the correlation between serum levels of acetate and the cardio-metabolic trait visceral fat. We found a strong negative association between both variables (Beta [95%CI] = −0.07 [−0.11, −0.04], *P* = 2.8 × 10^–4^) (Figure 2).

We then assessed the correlation between the acetate-associated gut genera and visceral fat. We found a strong positive correlation between *Lachnoclostridium* abundances and visceral fat (Beta [95%CI] = 0.076 [0.042, 0.11], *P* = 1.44 × 10^–5^). No significant associations were identified for the remaining five genera. We therefore conducted a formal mediation analysis to determine the indirect effects of acetate on the total effect of *Lachnoclostridium* on visceral fat. The analysis revealed that acetate acted as a potential partial mediator in the positive association between *Lachnoclostridium* and visceral fat (VAF = 10.3%, *P* = 2 × 10^–16^). These associations remained significant even after adjusting for dietary fiber intake.

Among the bacterial genera identified, we then genomically characterized *Lachnoclostridium* and *Coprococcus* because they presented the largest coefficient estimates in the association with acetate (Figure 2).

### Genomic-Based Taxonomic Classification and Phylogenetic Relationships of *Coprococcus* and *Lachnoclostridium* Species

The dendrograms created from the symmetric pairwise ANI values revealed the grouping of the 271 *Lachnoclostridium* and 1,121 *Coprococcus* genomes in 18 and 9 different species, respectively. Among them, most *Lachnoclostridium* species has been characterized (14 species), whereas *Coprococcus* presented four novel bacterial species and one has not been formally characterized so far.

In addition, we found that genomes identified as *C. sp900066115, C. sp00015424, C. sp. BIOML-A2, C. sp. BIOML-A1*, and *C. sp. NSJ-10* were assigned to the clusters of *C. eutactus, C. sp4* and *C. sp5* by the dendrogram. These misclassifications were further verified using their 16S rRNA sequences in a BLASTn search.

**FIGURE 3:**
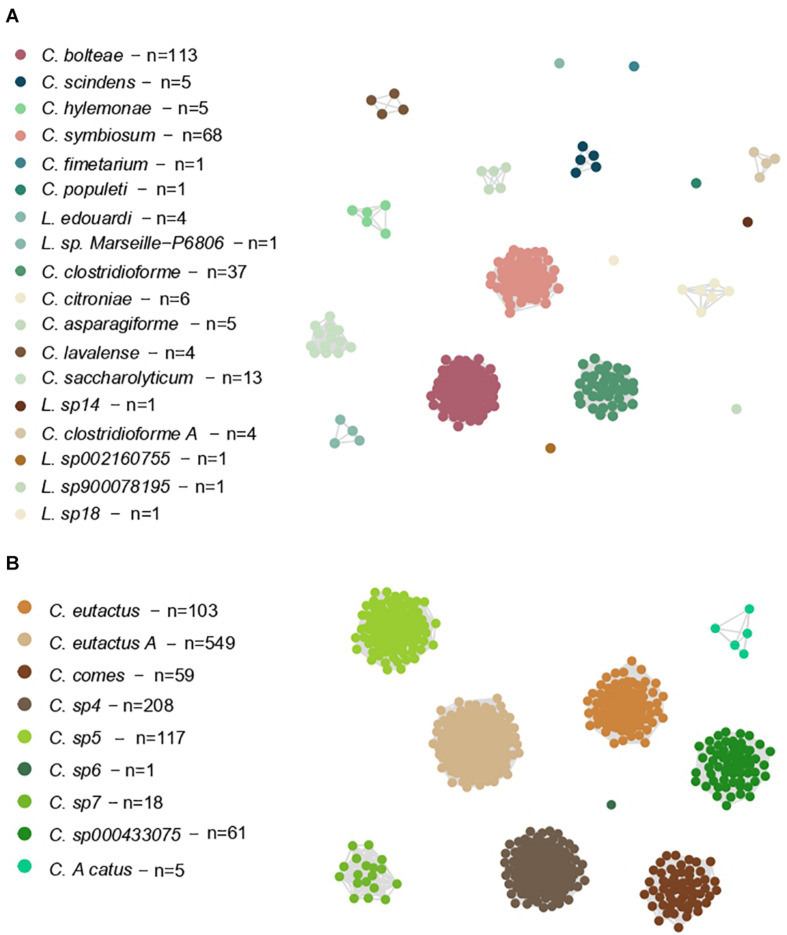
Weighted undirected graph based on symmetric pairwise ANI values of Lachnoclostridium **(A)** and Coprococcus **(B)**. Each node represents a single genome, and each edge represents a connection between two genomes that share a mean ANI value higher than 95%. Nodes are colored by the belonging Coprococcus and Lachnoclostridium species. The species that have not been formally characterized yet, have been named using “sp” followed by the cluster number given by the dendrogram. “n” indicates the genome number of each species.

We also constructed two networks based on these ANI values, allowing us to study the genomes as members of a connected system (Figure 3). Here, the largest clusters of *Lachnoclostridium* were shown by *C. bolteae* (113 genomes, 41% of the total), *C. symbiosum* (68 genomes, 25% of the total) and *C. clostridioforme* (37 genomes, 13% of the total), whereas *C. eutactus A* (549 genomes, 49% of the total), *C. sp4* (208 genomes, 19% of the total), *C. sp5* (117 genomes, 10% of the total) and *C. eutactus* (103 genomes, 9% of the total) presented the largest clusters of *Coprococcus.*

**FIGURE 4:**
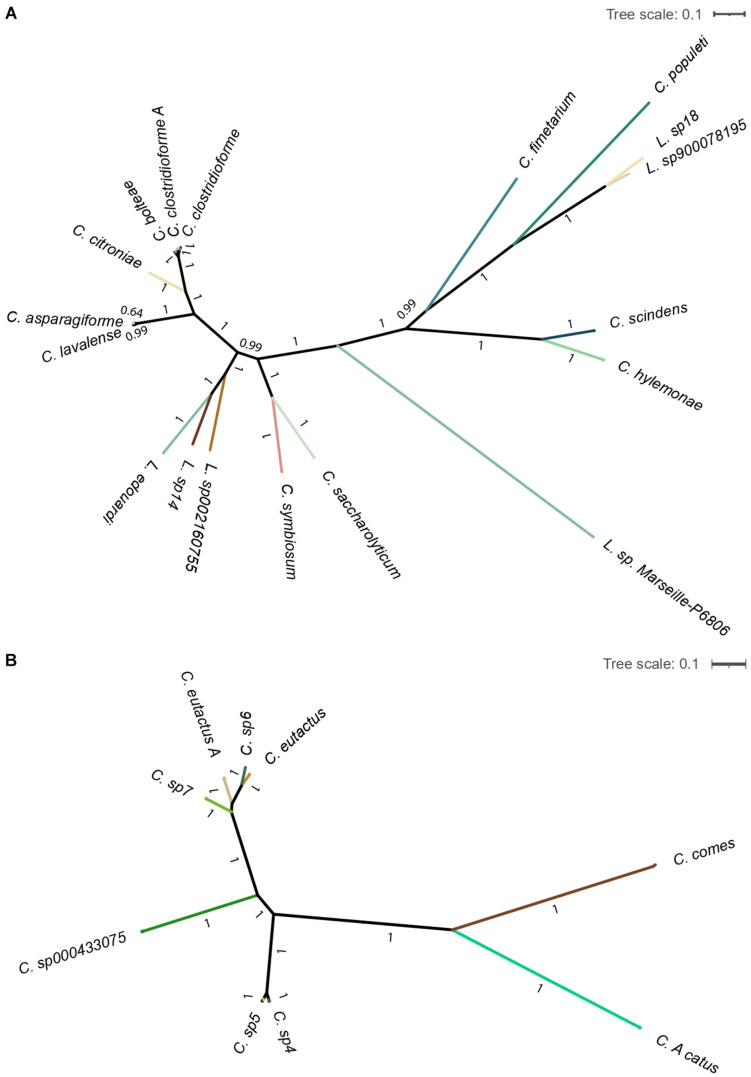
Maximum-likelihood phylogenetic tree of the genera Lachnoclostridium **(A)** and Coprococcus **(B)**. Tree is collapsed into clades at the species level and colored by species. Bootstrapping values for each branch are shown. The scale bar represents the average number of substitutions per site.

Additionally, the computed maximum-likelihood phylogenetic trees show the existing diversity of the gut bacteria *Lachnoclostridium* and *Coprococcus* (Figure 4). We observed that all the identified *Lachnoclostridium* and *Coprococcus* species represented well-defined independent lineages.

Of note, the shown *Clostridium* species in Part A of Figures 3, 4 belong to *Lachnoclostridium* (Yutin and Galperin, 2013).

### Prediction of the Functional Capabilities of *Coprococcus* spp. and *Lachnoclostridium* spp.

**FIGURE 5:**
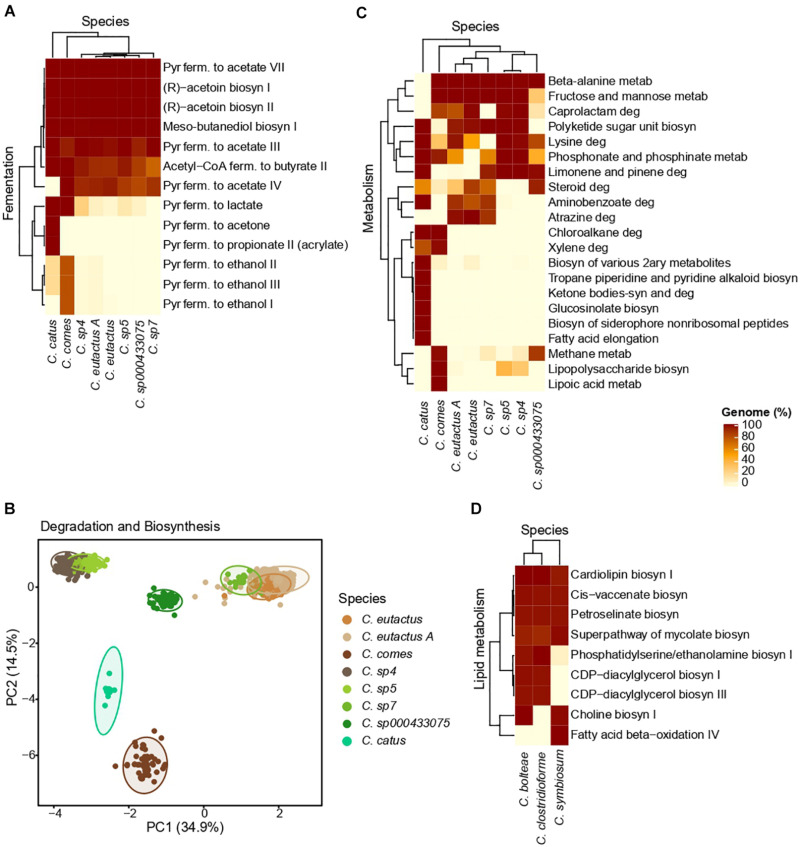
MetaCyc and KEGG results obtained for Coprococcus spp **(A–C)** and Lachnoclostridium spp **(D)**. **(A)** Heatmap of the MetaCyc fermentative pathways. **(B)** PCA clustering based on the MetaCyc data related to the biosynthesis and degradation processes of each species. **(C)** Heatmap of the KEGG metabolic pathways. Only the pathways highly differential (for a given pathway, at least one species has an abundance value <5% and another species has a value >80%) between species are shown. **(D)** Heatmap of the MetaCyc pathways related to lipid metabolism in the three major species of Lachnoclostridium (species with >15 genomes). Of note, the shown Clostridium species belong to Lachnoclostridium (Yutin and Galperin, 2013). For all the heatmaps, the pathways and species are hierarchically clustered, and color intensities represent the percentage of genomes of a given species with a specific metabolic pathway. Pyr, pyruvate; ferm., fermentation; metab, metabolism; deg, degradation; biosyn, biosynthesis.

The percentage of genomes in which a fermentative pathway was predicted in each *Coprococcus* species is depicted in Figure 5A. Genomes from all the *Coprococcus* species presented fermentative pathways involved in the acetate formation from pyruvate (range = 90–100% genomes), acetoin biosynthesis (100% genomes), butanediol biosynthesis (100% genomes) and butyrate formation from acetyl-CoA (range = 90–100% genomes). The pyruvate fermentation to acetone and propionate (acrylate pathway) was exclusively present in genomes from *C. catus* (100% genomes), whereas the production of ethanol from pyruvate was mainly found in genomes from *C. comes* (80% genomes). Production of lactate from pyruvate was predicted in the latter two species (100% genomes).

The PCA performed using the presence/absence matrix with the biosynthesis and degradation pathways show each species formed a well-defined cluster, being *C. sp4* and *C. sp5*, and *C. eutactus A, C. eutactus*, and *C. sp7* closely grouped (Figure 5B).

As shown in the heatmap of the KEGG metabolic pathways (Figure 5C), differences in the functional capabilities exit between *Coprococcus* species. For instance, beta-alanine metabolism was present in all the species (∼100% genomes), except in *C. catus*, whereas chloroalkene degradation was found only in *C. catus* and *C. comes* (100% genomes). On the other hand, some metabolic pathways were present in all the *Coprococcu* species in a percentage higher than 90% (Supplementary Table 5). The majority of them were related to the metabolism of amino acids such as alanine, aspartate, glutamate, arginine, proline, cysteine, glycine, serine and tyrosine, as well as essential amino acids such histidine, lysine, methionine, threonine, phenylalanine and tryptophan. Additionally, all the species presented the metabolism of several vitamins B, including vitamin B1 (thiamine), B2 (riboflavin), B3 (nicotinate), B6 (pyridoxine), B7 (biotin), and B9 (folate), and pathways involved in the carbohydrate metabolism, such as the pentose phosphate pathway and the starch and sucrose metabolism.

As we found a positive association between visceral fat and *Lachnoclostridium*, we focused on the metabolic pathways related to lipid metabolism. As depicted in Figure 5D, a high homogeneity in the functional capabilities related to lipid metabolism is presented in the three major species of *Lachnoclostridium*, above all between *C. bolteae* and *C. clostridioforme.* Furthermore, all the predicted pathways belong to the higher category of lipid biosynthesis, such as biosynthesis of choline I, CDP-diacylglycerol I and III, with the exception of the fatty acid beta-oxidation IV pathway, which belongs to the lipid degradation category. Moreover, genomes from the three major species presented pathways involved in the production of trimethylamine (TMA), including the biosynthesis of choline (∼100% genomes of *C. bolteae* and *C. symbiosum*) and phosphatidylethanolamine (∼100% genomes of *C. bolteae* and *C. clostridioforme*).

## Discussion

In what is to our knowledge the largest study to date investigating the associations of circulating acetate levels with gut microbiome composition and diversity and visceral fat, we report that circulating acetate levels are positively associated with microbiome alpha-diversity, while different gut bacterial genera are associated with either higher or lower acetate levels, and higher serum levels of acetate are correlated with lower visceral fat. We have also shown for the first time that the identified acetate-associated genus *Lachnoclostridium* has a strong positive correlation with visceral fat, and such association is partially mediated by acetate. Moreover, this is the first study genomically characterizing the acetate-associated gut genera *Lachnoclostridium* and *Coprococcus*, specifically, presenting their diversity and evolution at the genus level and annotating the functional capabilities of their species.

The identified positive associations between acetate and *Barnesiella* and *Ruminococcus* are consistent with the fact they contain genes involved in acetate production (Rey et al., 2010; Lustgarten,2019). We also found a negative correlation between acetate levels and *Bacteroides*. Strikingly, *Bacteroides* spp. are acetate producers (Miller, 1978; Robert et al., 2007). We speculate that a plausible reason why *Bacteroides* present a negative correlation is the co-presence of other bacteria that might utilize the acetate produced by *Bacteroides* to generate other metabolites.

Among the bacterial genera identified, we genomically characterized *Lachnoclostridium* and *Coprococcus* as they presented the largest coefficient estimates in the association with acetate, as well as the positive association between visceral fat and *Lachnoclostridium*. In addition, these two genera presented opposing effects on acetate levels, even though they are within the same family, and thus, their genomic characterisation can provide a more holistic perspective of the influence of the gut bacteria on human health.

The dendrogram of *Coprococcus* revealed that half of the identified species remained uncharacterized, indicating that *Coprococcus* is still a poorly known genus, whereas most *Lachnoclostridium* species presented less than six genomes suggesting that its members are very rare (low prevalence) or that may be present in the human gut at such extremely low abundances that are difficult to detect.

In addition, the dendrogram allowed us to identify misclassified genomes, emphasizing the importance of performing quality controls and taxonomic classification. We could further confirm that the groups of species obtained using an ANI threshold of 95% were correct, since all the identified species formed completely independent lineages. It is important to note that the bacterial species delineation was not affected by the high proportion of MAGs used (92% of *Coprococcus* genomes and 71% of *Lachnoclostridium* genomes from the total number). Therefore, the genomic methods proposed here can be generalizable to genomes from other bacterial species, independently of the genome type (reconstructed from metagenomes or sequenced from isolates).

Furthermore, to the best of our knowledge, the phylogenetic results represent the most complete overview of the phylogenetic relationships of species from the genera *Coprococcus* and *Lachnoclostridium* so far, as it includes non-characterized species.

The annotation of the fermentative pathways confirmed that the identified *Coprococcus* species present genes involved in the formation of acetate, explaining the found positive association between this genus and acetate. Moreover, *Coprococcus* species are known as butyrate producers (Pryde et al., 2002), supporting with our results, which show that the formation of butyrate was predicted in all species. Our results are also in line with the fact that *C. catus* can produce propionate via the acrylate pathway (Reichardt et al., 2014). *C. catus* and *C. comes* present fermentative pathways (e.g., ethanol and acetone production) which are not found in other species. Interestingly, these species clustered in a different clade in the phylogenetic tree at the genus level. Additionally, both might produce lactate. It is known that *C. comes* can also produce lactate and *C. catus* can produce propionate from this compound (Reichardt et al., 2014), however, *C. catus* is not recognized as a lactate producer. We hypothesize that the produced lactate in *C. catus* might be used to generate propionate or that this fermentative pathway is not active, as this genomic approach facilitates the prediction of the functional capabilities of this genus, but unable to infer active pathways.

When we analyzed the diversity of the functional capabilities related to the biosynthesis and degradation of compounds using a PCA, we observed a considerable functional diversity among species. Of note, *C. sp5* and *C. sp6*, and *C. eutactus A, C. eutactus* and *C. sp7* were closely clustered, again, these are closely related according to the phylogenetic tree, and thus, the lack of differences might be due to their evolutionary closeness. These results suggest that different species might be distinguished by their metabolic functional capabilities.

We also noted differences in several KEGG metabolic pathways between species. Some of these pathways have been associated with CMH. For instance, a higher aminobenzoate degradation has been associated with a body weight decrease (Pataky et al., 2016). Our results show that genomes from *C. catus*, *C. eutactus A, C. eutactus* and *C. sp7* might degrade aminobenzoate, and thus, positively influencing body weight.

Regarding the shared KEGG metabolic pathways, all the genomes presented starch and sucrose metabolism and pentose phosphate pathway, which are necessary to produce SCFAs (Topping and Clifton, 2001; Basen and Kurrer, 2020), and metabolism of essential amino acids, which can be absorbed meeting the amino acids requirements (Fuller and Tomé, 2005). Furthermore, all the species might be able to metabolize several vitamins/nutrients; including vitamins B, which has been associated with protective pathways involved in CMH; folate levels, which have been correlated with a lower metabolic syndrome score, plasma fasting glucose and a higher plasma HDL cholesterol (Navarrete-Muñoz et al., 2020); biotin, which has been shown to be involved in the glucose and lipid homeostasis (Fernandez-Mejia, 2005); thiamine, which may attenuate hypertension (Alaei-Shahmiri et al., 2015); and pyridoxine, might decrease triglyceride levels (Mottaghian et al., 2020).

Finally, we examined the lipid metabolism of the three major species of *Lachnoclostridium* as we found a positive association with visceral fat, as well as several studies have reported it to be related to diet-induced obesity (Zhao L. et al., 2017; Li et al., 2019; Sun et al., 2020), total cholesterol and LDL-C (Wang et al., 2020). Additionally, the mechanisms by through *Lachnoclostridium* impacts obesity remain unknown. Our results suggest that *Lachnoclostridium* spp. might negatively impact obesity and T2D. For instance, *Lachnoclostridium* spp might biosynthesize choline and phosphatidylethanolamine. Phosphatidylethanolamine can be methylated producing choline (Li and Vance, 2008), which can be subsequently used to produce TMA, and then trimethylamine-N-oxide (TMAO) in the liver (Zhu et al., 2018). This is in line with the fact that *Lachnoclostridium* has been suggested to be a TMA-producing bacteria (Jameson et al., 2016). Likewise, TMAO pathway has been associated with CMD in humans such as obesity and T2D (Dambrova et al., 2016; Schugar et al., 2017). Moreover, we identified in *C. bolteae* and *C. clostridioforme* two pathways involved in the biosynthesis of CDP-diacylglycerol, which might be a potential mediator of insulin resistance (Petersen and Shulman, 2018).

We are aware of some limitations in this study. The study sample includes only woman, and thus, our results might not be generalisable to men or different ranges of age. Only measures of acetate were available in this study, and therefore, we could not assess the associations between other relevant SCFAs, such as butyrate and propionate, and gut microbiota and visceral fat. These measures were performed using NMR, which provides different levels as compared to the gold standard LC-MS methodology. Furthermore, the association study was performed using 16S rRNA gene sequencing data. Our findings would have benefited from metagenomic sequencing analyses and an independent dataset to replicate our results or in vitro demonstrations.

Notwithstanding the above limitations, we have shown for the first time that higher abundances of *Lachnoclostridium* lead to lower circulating levels of acetate, resulting in increasing visceral fat. In addition, *Coprococcus* may play an important role in host health by its production of vitamins B and SCFAs, whereas *Lachnoclostridium* might have a negative impact on CMH by influencing negatively the circulating levels of acetate and being involved in the biosynthesis of harmful lipid compounds, such as TMA and CDP-diacylglycerol. We have also presented a dataset that compiles 271 and 1,121 high-quality genomes of *Lachnoclostridium* and *Coprococcus*, respectively, which can be very useful for scientists working in this area.

## Data Availability Statement

16S sequencing data used for this study is deposited in the European Nucleotide Archive (ERP015317). All other TwinsUK data are available upon request on the department website (http://www.twinsuk.ac.uk/dataaccess/accessmanagement/). All the metagenome data generated during the current study are included in the Supplementary Material.

## Ethics Statement

Twins provided informed written consent and the study was approved by St. Thomas’ Hospital Research Ethics Committee (REC Ref: EC04/015).

## Author Contributions

CM and AMV conceived and designed the experiments. AN analyzed the data. PL, XZ, PMW, CJS, TDS, and MF contributed reagents/materials/analysis tools. AN drafted the first version of the manuscript. AMV and CM edited and revised the manuscript. All authors have readthe final manuscript and approved it for publication.

## Conflict of Interest

TDS is co-founder of Zoe Global Ltd. AMV is a consultant for Zoe Global Ltd. The remaining authors declare that the research was conducted in the absence of any commercial or financial relationships that could be construed as a potential conflict of interest.

## Supplementary Material

Supplementary Table 1 The created dataset containing 1,121 ultra high-quality genomes belonging to *Coprococcus* and 271 high-quality genomes belonging to *Lachnoclostridium* and their respective metadata.

Supplementary Tables 2, 3, 4 Average nucleotide identity (ANI) values obtained for each pair of genomes belonging to *Lachnoclostridium* and *Coprococcus* using FastANI. Previously, genomes were filtered by alignment fraction (>0.4).

Supplementary Table 5 KEGG and MetaCyc pathways obtained for *Coprococcus* and *Lachnoclostridium* genomes along with the percentage of genomes of each species presenting a given pathway.

## Abbreviations

ANI: average nucleotide identity
ASV: amplicon sequence variants
CMD: cardio-metabolic diseases
CMH: cardio-metabolic health
CVD: cardiovascular diseases
KEGG: Kyoto Encyclopedia of Genes and Genomes
MAG: metagenome-assembled genomes
MetaCyc: Metabolic Pathway Database
NCBI: National Center for Biotechnology Information
SCFA: short-chain fatty acids
T2D: type-2 diabetes
TMA: trimethylamine
TMAO: trimethylamine-N-oxide
UHGG: Unified Human Gastrointestinal Genome.
